# Supplementary material for: tagPAINT: covalent labelling of genetically encoded protein tags for DNA-PAINT imaging
Source: R Soc Open Sci. 2019 Dec 11;6(12):191268. doi: 10.1098/rsos.191268 (PMC6936279; doi:10.1098/rsos.191268)
Supplement: Figures S1 and S2 [file rsos191268supp1.pdf]

tagPAINT: covalent labelling genetically encoded protein tags for DNA-PAINT imaging

Daniel J. Nieves[1,2,3], Geva Hilzenrat[2,3,4], Jason Tran[2,3], Zhengmin Yang[2], Hugh H. MacRae[2], Matthew A. B. Baker[5], J. Justin Gooding[6] & Katharina Gaus[2,3*]

[1]Institute of Immunology and Immunotherapy, College of Medical and Dental Sciences, University of Birmingham, Birmingham, B15 2TT, UK.
[2]EMBL Australia Node in Single Molecule Science, School of Medical Sciences, University of New South Wales, Sydney, Australia.
[3]ARC Centre of Excellence in Advanced Molecular Imaging, University of New South Wales, Sydney, Australia.
[4] Commonwealth Scientific and Industrial Research Organisation (CSIRO), Manufacturing, Clayton, VIC 3168, Australia.
[5]School of Biotechnology and Biomolecular Science, University of New South Wales, Sydney, NSW 2052, Australia.
[6]School of Chemistry, Australian Centre for NanoMedicine and the ARC Centre of Excellence in Convergent Bio-Nano Science and Technology, University of New South Wales, Sydney, Australia.
*Correspondence: k.gaus@unsw.edu.au

SUPPLEMENTARY FIGURES

SUPPLEMENTARY FIGURES

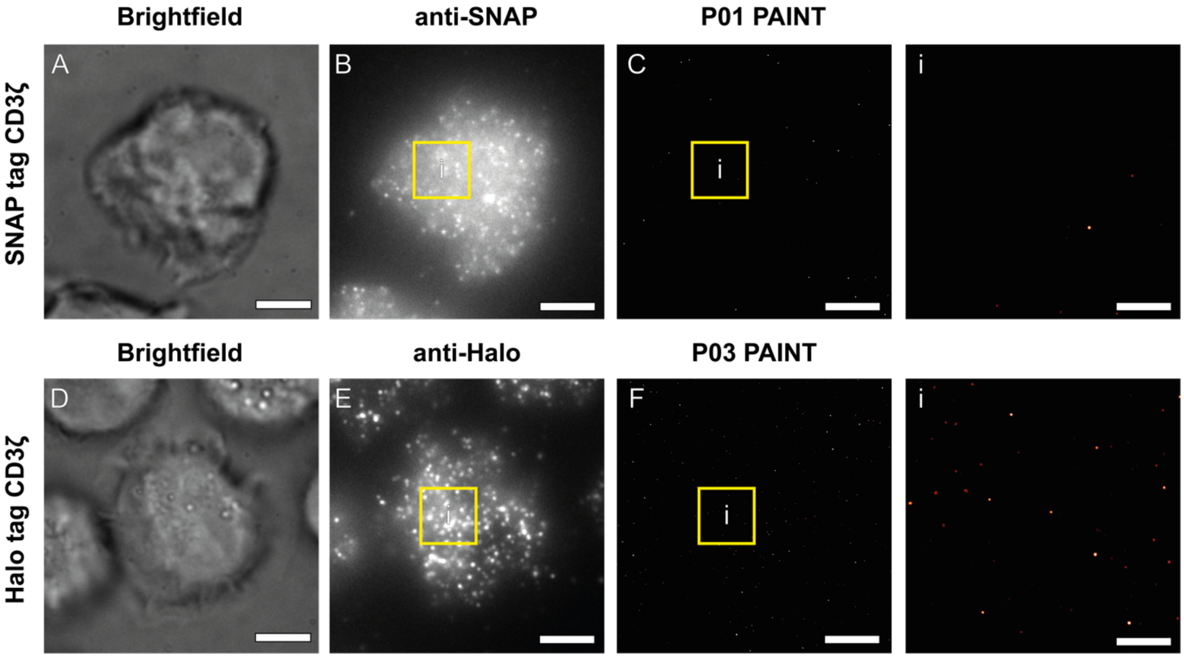

Figure S1. Background from tagPAINT imaging in the absence of tag ligand labelling. CD3ζ KO Jurkat cells expressing SNAP- CD3ζ (**A**-**C**) and Halo- CD3ζ (**D-F**) were processed and imaged using DNA-PAINT as in Figure 1, however, the ligand for the SNAP and Halo tags was not incubated with the cells prior to imaging. **A**) Brightfield of CD3ζ KO Jurkat cells expressing SNAP- CD3ζ. **B**) Anti-SNAP-ATTO488 staining. **C**) DNA-PAINT imaging (2nM, P01 ATTO655 imager), with zoomed region from the centre of the cell (i, yellow box) shown (right). **D**) Brightfield of CD3ζ KO Jurkat cells expressing Halo- CD3ζ. **B**) Anti-Halo-ATTO568 staining. **C**) DNA-PAINT imaging (2nM, P03 ATTO655 imager), with zoomed region from the centre of the cell (i, yellow box) shown (right). Scale bars for all larger images are 5 μm and for zoomed images 1μm.

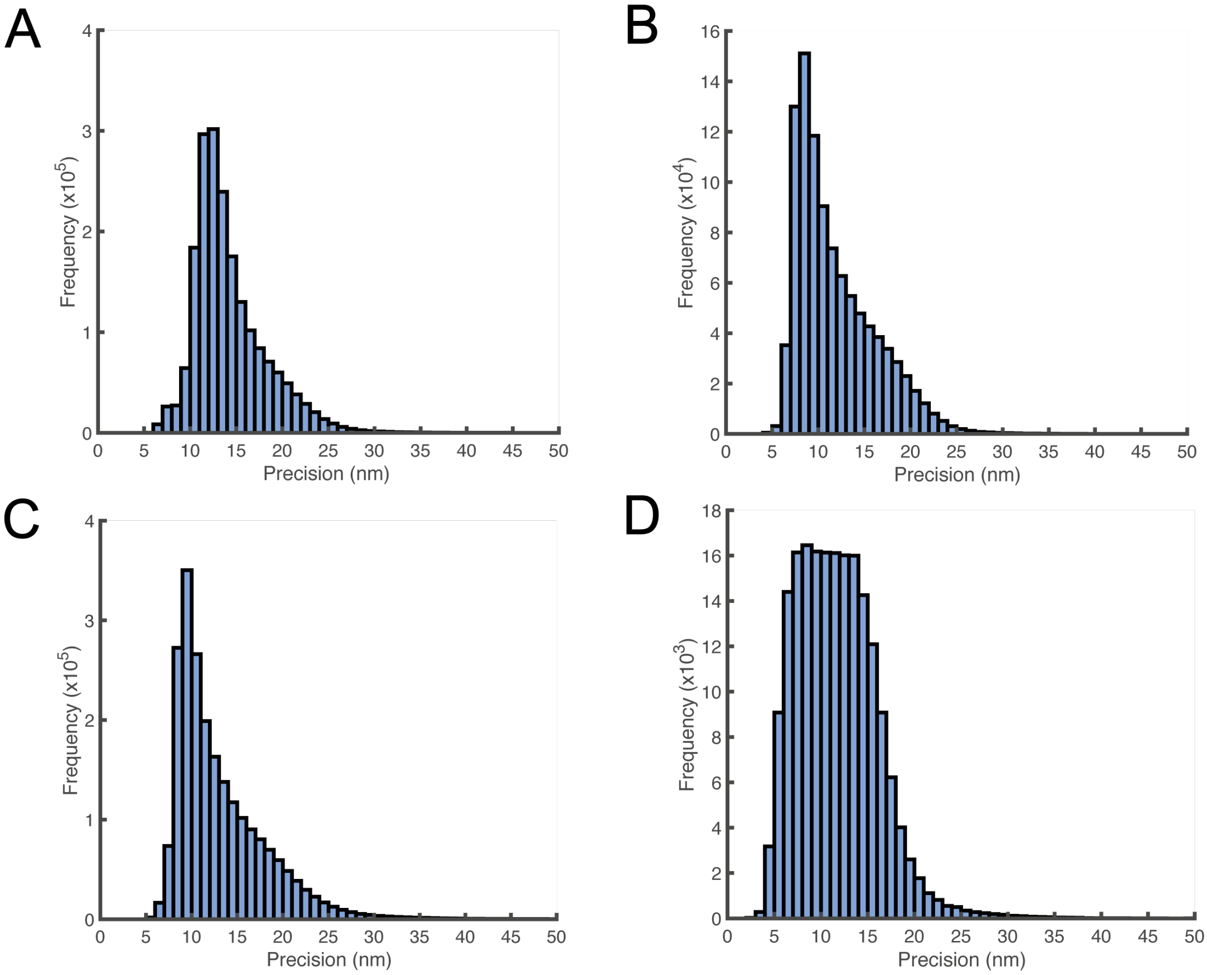

Figure S2. Localisation precision histograms of tagPAINT imaging from Figure1. The localisation precision was calculated for each event within the image acquisition according to Mortensen *et al*. **A**) Localisation data from LAT-SNAP tagPAINT data (Figure 1A). **B**) Localisation data from CD3ζ-SNAP tagPAINT data (Figure 1B). **C**) Localisation data from LAT-Halo tagPAINT data (Figure 1C). **D**) Localisation data from CD3ζ-Halo tagPAINT data (Figure 1D).
